# Migration background, oral hygiene behavior and oral health literacy: findings based on a quota-based sample

**DOI:** 10.3389/froh.2026.1791168

**Published:** 2026-04-23

**Authors:** Ghazal Aarabi, Berit Lieske, Tjore Model, Hans-Helmut König, André Hajek

**Affiliations:** 1 Department of Periodontics, Preventive and Restorative Dentistry, Center for Dental and Oral Medicine, University Medical Center Hamburg-Eppendorf, Hamburg, Germany; 2 Department of Health Economics and Health Services Research, University Medical Center Hamburg-Eppendorf, Hamburg Center for Health Economics, Hamburg, Germany

**Keywords:** duration of tooth brushing, electric toothbrush, fluoride gel, interdental cleaning aids, migration background, oral health, oral health literacy, oral hygiene behavior

## Abstract

**Aim:**

To describe oral hygiene behavior and oral health literacy in the general adult German population (also stratified by migration background) and to identify the association of migration background with such outcomes.

**Methods:**

Data from the general adult German population aged 18–74 years was used (quota-based sample representative in terms of age, gender, and federal state). The analytic sample equaled 5,000 individuals, with mean age of 47 years, 51% being female, and 11% had a migration background. Common questions were employed to measure oral hygiene behavior, oral health literacy and migration background. Multiple logistic regressions were used to explore the association of migration background with oral hygiene behavior and oral health literacy.

**Results:**

A significant proportion reported not using oral hygiene products, with 35.5% not using an electric toothbrush (not using manual toothbrush: 28.1%), 59.4% not using fluoride gel, and 27.7% not using interdental cleaning aids. Additionally, 14.1% brushed their teeth for less than two minutes and 76.4% reported having at least good oral health literacy. Depending on migration background, some outcomes significantly varied. Adjusted multiple logistic regressions showed that having a migration background (compared to not having a migration background) was significantly associated with lower odds of using an electric toothbrush (OR = 0.79, 95% CI:0.66–0.95) and duration of tooth brushing (OR = 0.54, 95% CI:0.43–0.69).

**Conclusion:**

Data on oral health, use of different oral hygiene products, and oral health literacy among adults with migration background is limited. Our analysis adds to this knowledge and helps identify gaps and needs in this population to improve preventive measures.

## 1 Introduction

Maintaining good oral hygiene, including regular tooth brushing, use of interdental cleaning products, and regular visits to the dentist, is essential for preventing and managing oral health problems. Since the Third German Oral Health Study (DMS) in 1997, toothbrushing patterns have continuously improved among the German population (1). According to recent data from the Sixth DMS (DMS 6), currently 82.2% of the adult (35- to 44-year-olds) and 84.0% of the older adult (65- to 74-year-olds) participants brush their teeth ≥2 times daily, with more than half (55.4% for adults; 51.0% for older adults) using an electric toothbrush or both electric and manual (2). The oral health behavioral targets of the oral health goals for Germany 2030 (3) include increasing the proportion of twice-daily toothbrushing to 85.3% and 89.1% for adults and seniors, respectively. In addition to better oral health behaviors, greater knowledge on oral health is also associated with better oral health outcomes. Yet, only a few studies have examined oral health literacy among the German adult population (4–6). For example, the Health Literacy Survey Germany 2 (HLS-GER 2), a representative quantitative survey of the German-speaking resident population aged ≥18, demonstrated that 58.8% of the participants displayed low health literacy (4). Additionally, according to the Hamburg-based MuMi intervention study (*Promotion of oral health and oral health literacy of people with migration background*), only 17% of the participants self-reported having very good or excellent knowledge of oral health issues (5).

While education, smoking or/and other factors have been identified as well-established risk factors for oral health outcomes (7–10), recent discussions have also focused on migration background. The results of the DMS 6 demonstrate that the frequency of tooth brushing as well as interdental cleaning differed significantly among adults and older adults without and with migration background (11). The latter overall presented poorer oral hygiene behavior and a more complaint-oriented utilization of dental services. Furthermore, with regard to oral health literacy, participants of the MuMi study with migration background presented significantly lower oral health literacy [measured with the OHLP (12)] compared to those participants without migration background (5).

Due to the restricted knowledge, the current study aims to describe oral hygiene behavior and oral health literacy in the general adult German population and to identify the association of migration background with such outcomes.

## 2 Materials and methods

### 2.1 Sample

Data from a large sample of 5,000 participants aged 18–74 in Germany were collected during August and September 2023. Recruitment of participants was performed by Bilendi (a market research company certified under ISO 26362). To ensure a representative sample that reflects the demographic diversity of the German adult population, participants were selected from an online pool based on a quota sampling approach (age, gender, and federal state). Notably, because our sample matched the target population in terms of age, gender, and federal state, sampling weights were not needed

Prior to participation, all individuals provided their informed consent. The study was approved by the Local Psychological Ethics Committee at the University Medical Centre Hamburg-Eppendorf (LPEK-0629).

### 2.2 Outcomes: oral hygiene behavior and oral health literacy

Respondents were asked how often they use the following products (1 = not at all; 2 = once a week or less; 3 = at least twice a week; 4 = at least once a day; 5 = at least twice a day):

- Use of an electric toothbrush
- Use of a manual toothbrush
- Use of fluoride gel
- Use of interdental cleaning aids (dental floss and/or interdental brushes)

Furthermore, individuals were asked how long they usually brush their teeth (1 = about half a minute or less; 2 = about one minute; 3 = about two minutes; 4 = about three minutes; 5 = about four minutes or more). Moreover, individuals were asked about their oral health literacy (single-item tool; answer categories: 1 = excellent; 2 = very good; 3 = good; 4 = moderate; 5 = poor; detailed evaluation criteria were not provided). Thus, in total, seven outcomes were used in this study (frequency of use of electric toothbrush; frequency of use of manual toothbrush; frequency of use of fluoride gel; frequency of use interdental cleaning aids; duration of tooth brushing; oral health literacy). It should be emphasized that the outcomes are based on self-reports.

For descriptive purposes, the metric outcomes were retained. These were then dichotomized as follows for the regression analyses: 1 if frequency of use of electric toothbrush at least once a day (0 otherwise), 1 if frequency of use of manual toothbrush at least once a day (0 otherwise), 1 if frequency of use of fluoride gel once a week (0 for all other response options) (13), 1 if frequency of use of interdental cleaning aids at least once a day (0 otherwise) (13, 14), 1 if duration of tooth brushing at least two minutes (0 otherwise), 1 if oral health literacy at least good (0 otherwise).

### 2.3 Migration background

Defined by the German Federal Statistics Office, whoever was born as a non-German citizen or born to at least one parent without German citizenship has a “migration background” (13). In the questionnaire, migration background was quantified based on a self-rating (no or yes) to the question: “Do you have a migration background? A person has a migration background if he or she or at least one parent was not born with German citizenship”.

### 2.4 Covariates

Several covariates were included in the regression analysis based on previous research (5, 11, 14). Sociodemographic covariates included age, gender (men; women; diverse), and education [based on the CASMIN classification proposed by Brauns and Steinmann (15): primary education, secondary education, and tertiary education]. Moreover, the lifestyle-related covariate smoking habits (four levels from “never smoked” to “daily smoker”) was used. In terms of health-related covariates, self-rated health [measured on a scale from very poor (1) to very good (5)] was included.

### 2.5 Statistics

First, sample characteristics are described (total sample and stratified by migration background). Then, the oral hygiene behavior and oral health literacy were described (for the total sample and stratified by migration background). Additionally, the outcomes were presented stratified by gender, age group, and education in further analysis. After that, the association of having a migration background with oral hygiene behavior and oral health literacy was explored using unadjusted and adjusted logistic regressions. We also calculated variance inflation factors (VIFs). However, they were low (mean VIF was 1.08 and highest VIF was 1.18), indicating that multicollinearity is not a threat.

There were no missing values because the forced-choice format ensured complete responses. For the statistical analysis, we used StataNow 19.5 (Stata Corp., College Station, Texas), and statistical significance was determined at a threshold of *p* < 0.05.

## 3 Results

### 3.1 Sample characteristics

Sample characteristics (among the total sample and stratified by migration background) are presented in Table 1. In the total sample, average age equaled about 47 years (SD: 15 years; 18–74 years), with 50.8% being female. Individuals with and without migration background statistically significantly differed in all variables. Further details are presented in Table 1.

**Table 1:** Sample characteristics—stratified by migration background and among the total sample.

Variables	Individuals without migration background (*n* = 4,449, 89.0%)	Individuals with migration background (*n* = 551, 11.0%)	Total sample (*n* = 5,000, 100%)	*P*-value
Gender: N (%)				0.04
Men	2,196 (49.4)	255 (46.3)	2,451 (49.0)	
Women	2,247 (50.5)	293 (53.2)	2,540 (50.8)	
Diverse	6 (0.1)	3 (0.5)	9 (0.2)	
Age (years): Mean (SD)	47.9 (15.1)	38.8 (14.1)	46.9 (15.3)	<0.001
Education: N (%)				0.01
Primary education	481 (10.8)	52 (9.4)	533 (10.7)	
Secondary education	2,682 (60.3)	305 (55.4)	2,987 (59.7)	
Tertiary education	1,286 (28.9)	194 (35.2)	1,480 (29.6)	
Smoking status: N (%)				<0.001
Yes, daily	1,059 (23.8)	125 (22.7)	1,184 (23.7)	
Yes, occasionally	430 (9.7)	86 (15.6)	516 (10.3)	
No, not anymore	1,255 (28.2)	111 (20.1)	1,366 (27.3)	
Never smoked before	1,705 (38.3)	229 (41.6)	1,934 (38.7)	
Self-rated health (1 = very poor to 5 = very good): Mean (SD)	3.6 (0.8)	3.8 (0.8)	3.6 (0.8)	<0.001

### 3.2 Description of oral hygiene behavior and oral health literacy

Oral hygiene behavior for the total analytic sample of *n* = 5,000 (and additionally stratified by migration background) is displayed in Table 2. In the total sample, 35.5% (28.1%) of the respondents did not use an (manual) electric toothbrush. Moreover, 59.4% of the respondents did not use fluoride gel, and 27.6% of the respondents did not use interdental cleaning aids. About 44.6% of the respondents brushed their teeth for about two minutes. Additionally, 76.4% of the respondents reported having at least good oral health literacy. Bivariately, migration background was significantly associated with frequency of use of electric toothbrush, frequency of use of manual toothbrush, frequency of use of interdental cleaning aids, and duration of tooth brushing. More details are shown in Table 2.

**Table 2:** Oral hygiene behavior and oral health literacy—stratified by migration background and among the total sample.

Oral hygiene behavior	Individuals without migration background (*n* = 4,449, 89.0%)	Individuals with migration background (*n* = 551, 11.0%)	Total sample (*n* = 5,000, 100%)	*P*-value
Frequency of use of electric toothbrush: N (%)				<0.001
Not at all	1,570 (35.3)	204 (37.0)	1,774 (35.5)	
Once a week or less	183 (4.1)	47 (8.5)	230 (4.6)	
At least twice a week	207 (4.7)	22 (4.0)	229 (4.6)	
At least once a day	1,016 (22.8)	104 (18.9)	1,120 (22.4)	
At least twice a day	1,473 (33.1)	174 (31.6)	1,647 (32.9)	
Frequency of use of manual toothbrush: N (%)				<0.01
Not at all	1,288 (29.0)	119 (21.6)	1,407 (28.1)	
Once a week or less	506 (11.4)	74 (13.4)	580 (11.6)	
At least twice a week	288 (6.5)	46 (8.3)	334 (6.7)	
At least once a day	1,056 (23.7)	145 (26.3)	1,201 (24.0)	
At least twice a day	1,311 (29.5)	167 (30.3)	1,478 (29.6)	
Frequency of use of fluoride gel: N (%)				0.13
Not at all	2,657 (59.7)	311 (56.4)	2,968 (59.4)	
Once a week or less	861 (19.4)	99 (18.0)	960 (19.2)	
At least twice a week	328 (7.4)	52 (9.4)	380 (7.6)	
At least once a day	337 (7.6)	46 (8.3)	383 (7.7)	
At least twice a day	266 (6.0)	43 (7.8)	309 (6.2)	
Frequency of use of interdental cleaning aids: N (%)				<0.001
Not at all	1,270 (28.5)	108 (19.6)	1,378 (27.6)	
Once a week or less	809 (18.2)	120 (21.8)	929 (18.6)	
At least twice a week	867 (19.5)	118 (21.4)	985 (19.7)	
At least once a day	1,012 (22.7)	136 (24.7)	1,148 (23.0)	
At least twice a day	491 (11.0)	69 (12.5)	560 (11.2)	
Duration of tooth brushing: N (%)				0.02
About half a minute or less	94 (2.1)	18 (3.3)	112 (2.2)	
About one minute	509 (11.4)	85 (15.4)	594 (11.9)	
About two minutes	2,006 (45.1)	225 (40.8)	2,231 (44.6)	
About three minutes	1,420 (31.9)	171 (31.0)	1,591 (31.8)	
About four minutes or more	420 (9.4)	52 (9.4)	472 (9.4)	
Oral health literacy: N (%)				0.10
Excellent	235 (5.3)	43 (7.8)	278 (5.6)	
Very good	962 (21.6)	127 (23.0)	1,089 (21.8)	
Good	2,200 (49.4)	253 (45.9)	2,453 (49.1)	
Moderate	978 (22.0)	117 (21.2)	1,095 (21.9)	
Poor	74 (1.7)	11 (2.0)	85 (1.7)	

Supplementary Tables S1, S2 and S3 showed oral hygiene behavior and oral health literacy stratified by gender, age group, and education, respectively. Depending on such sociodemographic variables, significant differences were present for (nearly) all outcomes. Additional details are depicted in Supplementary Tables S1, S2 and S3.

### 3.3 Regression analysis

Unadjusted (Table 3) and adjusted (Table 4) logistic regressions are displayed. Several fit statistics are shown in Supplementary Table S4. In unadjusted regression analysis, having a migration background (compared to not having a migration background) was significantly associated with lower odds of using an electric toothbrush (OR = 0.80, 95% CI:0.67–0.96), and duration of tooth brushing (OR = 0.68, 95% CI:0.54–0.86). The findings of adjusted regressions were comparable to those based on unadjusted regressions. More precisely, having a migration background (compared to not having a migration background) was significantly associated with lower odds of using an electric toothbrush (OR = 0.79, 95% CI: 0.66–0.95) and duration of tooth brushing (OR = 0.54, 95% CI:0.43–0.69).

**Table 3:** Association of migration background with oral hygiene behavior and oral health literacy. Results based on unadjusted logistic regressions.

	(1)	(2)	(3)	(4)	(5)	(6)
Independent variables	Use of electric toothbrush	Use of manual toothbrush	Use of fluoride gel	interdental cleaning aids	Duration of tooth brushing	Oral health literacy
Migration background: Yes (Reference category: No)	0.80*	1.15	0.91	1.16	0.68**	1.02
	(0.67–0.96)	(0.96–1.37)	(0.73–1.15)	(0.97–1.40)	(0.54–0.86)	(0.83–1.26)
Constant	1.27***	1.14***	0.24***	0.51***	6.38***	3.23***
	(1.20–1.35)	(1.07–1.21)	(0.22–0.26)	(0.48–0.54)	(5.85–6.95)	(3.01–3.46)
Observations	5,000	5,000	5,000	5,000	5,000	5,000
Pseudo R-squared	0.001	0.0003	0.00001	0.0004	0.002	0.0000

Odds Ratios are reported, 95% CI in parentheses, *** *p* < 0.001, ** *p* < 0.01, * *p* < 0.05,+*p* < 0.10.

**Table 4:** Association of migration background with oral hygiene behavior and oral health literacy. Results based on adjusted logistic regressions.

	(1)	(2)	(3)	(4)	(5)	(6)
Independent variables	Use of electric toothbrush	Use of manual toothbrush	Use of fluoride gel	interdental cleaning aids	Duration of tooth brushing	Oral health literacy
Migration background: Yes (Reference category: No)	0.79*	1.11	0.79+	1.19+	0.54***	0.92
	(0.66–0.95)	(0.93–1.34)	(0.63–1.00)	(0.99–1.44)	(0.43–0.69)	(0.74–1.14)
Covariates	✓	✓	✓	✓	✓	✓
Constant	0.25***	1.35	0.35***	0.07***	7.77***	0.29***
	(0.17–0.39)	(0.89–2.04)	(0.21–0.60)	(0.04–0.11)	(4.33–13.92)	(0.18–0.46)
Observations	5,000	5,000	5,000	5,000	5,000	5,000
Pseudo R-squared	0.02	0.007	0.020	0.026	0.026	0.044

Odds Ratios are reported, 95% CI in parentheses, *** *p* < 0.001, ** *p* < 0.01, * *p* < 0.05,+*p* < 0.10; Covariates include age, gender, education, smoking, and self-rated health.

## 4 Discussion

We aimed to reveal oral hygiene behavior and oral health literacy in Germany (also stratified by migration background). Moreover, we aimed to reveal the potential link of migration background with the aforementioned outcomes. Adjusted regressions showed that having a migration background was significantly associated with lower odds of using an electric toothbrush and duration of tooth brushing.

Since the late 1980s, the DMS has reported on oral health status and behavior of the German population (1, 2, 16–19). Compared to 82.2% among the adults (35- to 44-year-olds) and 84.0% among the older adults (65- to 74-year-olds) of the DMS 6 (2), at 85.8% our study participants, aged 47 years on average, demonstrated a similar toothbrushing frequency of at least two minutes. In addition to a higher frequency, a longer brushing time leads to increased biofilm reduction (22–25). According to a systematic review by Slot et al. (22), brushing for 1 min reduces plaque index values by 27%, while brushing for 2 min reduces them by 41%. Additionally, in a randomized controlled trial on the effect of toothbrushing time on dental plaque removal *in vivo*, the authors found that extending the brushing time to two to three minutes led to a 55% increase in biofilm reduction compared to brushing for 30 s (23). The DMS 6 furthermore reports data on the type of toothbrush (electric or manual) used, yet data on the frequency of use (e.g., at least twice daily) is not reported. Our data shows that more than one-fifth (22.4%) and almost a quarter (24%) of the participants use an electric or manual toothbrush at least twice a day, respectively. There have been discussions on the efficacy of electric vs. manual toothbrushes for plaque removal. While some studies show greater efficacy of electric toothbrushes for plaque and gingivitis reductions (20–22), the consensus is that the duration and frequency of use as well as the systematics in toothbrushing is most relevant for how effectively the teeth are cleaned. Instructions on systematic brushing represents an option for improving brushing efficiency (29). Admittedly, data on how effectively people brush their teeth at the population level is limited. Future evaluations within the DMS 6, however, will include video-analyses of people's brushing techniques.

The use of fluoride gel on caries prevention has additionally been shown through several studies, including systematic reviews (23–27). For example, in a systematic review with meta-analysis of data from various studies, Marinho et al. (33) conclude that there is moderate evidence for a significant caries-preventive effect. This effect is independent of the application of other fluoridation measures or the type of gel application (professional with an applicator or brushing at home). Yet, the prevalence of its use is almost never reported. Overall, while the majority of studies focused solely on toothbrushing frequency and which toothbrush was used, our survey further reports on the use of additional oral hygiene products as well as the frequency of use. Although the benefits of a daily oral hygiene regimen, including interdental brushes and dental floss, on oral health, particularly gingivitis, have been established (28–31), reports on the prevalence of the use of such products is rare. In a study by Knöfler et al. in Lower Saxony, Germany, 38% and 5% of laypersons of a survey-based cross-sectional study reported using dental floss and interdental brushes, respectively (32). However, information on the frequency of use was not reported. Our results show, around 23% of our study participants used either dental floss or interdental brushes at least once daily. Inter-dental cleaning is essential in order to maintain interproximal gingival health, provides higher levels of plaque removal than toothbrushing alone, and should be incorporated on a routine basis when managing inflammation (33). As such, recent 7-year follow-up data from the Study of Health in Pomerania (SHIP-TREND) revealed a reduction in tooth loss incidence among interdental cleaning aid users and a 32% lesser likelihood of having higher interdental plaque compared to non-users (34).

Our sample was additionally stratified by migration background. In 2024, approximately 30% of the German population had a migration background (35). Research indicates that immigrants and their children vary in their susceptibility to diseases, their available resources to manage these risks, and their access to healthcare services (36). In our adjusted regression model, we found that having a migration background (compared to not having a migration background) was significantly associated with lower odds of using an electric toothbrush (OR = 0.79, 95% CI:0.66–0.95) and the duration of tooth brushing (OR = 0.54, 95% CI:0.43–0.69). Cross-sectional data of the DMS 6 (14) supports this by showing that significantly less adult participants with migration background brushed their teeth ≥2 min compared to those without migration background (73.9% vs. 85.1%). Moreover, after adjusting for confounders (age, sex, education), association analyses revealed migration background as an independent risk factor for poorer oral health outcomes (14). Furthermore, the MuMi study (5) also found lower toothbrushing frequencies among those participants with migration background. Apart from that, the authors additionally demonstrated in linear mixed models that those with migration background performed significantly worse in their oral health literacy scores, even after adjusting for several covariates (sex, age, education) (5). Similar trends were found in other studies globally (37). Although not significant, the results of our adjusted model demonstrate lower odds for oral health literacy among participants with migration background. In contrast to our study, oral health literacy in the MuMi study was assessed with a validated multi-item questionnaire that assesses different dimensions of oral health literacy in more detail (oral health knowledge in general, health care knowledge, and oral hygiene behavior) (12), which might account for the differences. Importantly, limited oral health literacy is associated with poorer oral hygiene behaviors, therefore increasing the risk for poorer oral health outcomes (5, 38, 39). Consequently, greater awareness, better knowledge, and more positive attitudes toward oral hygiene and care are essential components of overall oral health outcomes that should be specifically promoted as part of preventive measures to minimize oral health problems (48).

It is important to highlight both the strengths and limitations of this study. This research is one of the first studies describing oral hygiene behavior in the German adult population with and without migration background in greater detail. Data were drawn from a quota-based sample of 5,000 individuals (ensuring representativeness regarding age, gender, and federal state). The outcomes were quantified using items with high face validity.

It is important to mention that the variable migration background was quantified based on the basis of a single self-reported question [“Do you have a migration background?” (yes or no)] and not calculated based on participants' details about their own place of birth or that of their parents (based on register data) (40). While this is common practice for large-scale surveys, this may oversimplify a population that is inherently heterogeneous. Linguistic, structural, and socioeconomic differences as well as additional migration-related determinants (such as length of stay or origin) must be taken into account in order to understand the complexity and variability of oral health among migrant communities (49, 50). This reiterates the need for more nuanced categorizations and culturally sensitive approaches in future studies.

Furthermore, we must highlight that the findings are based on cross-sectional data, which limits the discovery of a causal link. While the sample is representative in the aforementioned terms, only people with sufficient German language skills were able to participate in the survey, which could limit the generalizability within the heterogeneous German population, especially when including migration background as independent variable. It should be noted that oral health literacy in this study was based on a single-item self-report by the study participants, not measured using a validated multi-item oral health literacy instrument (12, 51). While the choice might be understandable considering practical constraints (e.g., survey length, respondent burden), single-item measures might lack psychometric properties and sensitivity and overestimate perceived knowledge (52, 53). Generally, self-reports can introduce some bias (e.g., social desirability bias or recall bias). Additionally, unmeasured factors (e.g., linguistic barriers, economic hardship, access to dental care, or health insurance status) could affect our estimates and thus should be included in future studies.

## 5 Conclusion

Data on oral health, particularly detailed information on the use of different oral hygiene products, and oral health literacy among adults with migration background, is currently limited to a few studies. Thus, our current analysis adds to the limited knowledge that is currently available for this population. Since proper oral hygiene is key in maintaining good oral health, providing targeted culture-sensitive information on oral hygiene practices and behaviors for these populations could aid in improving preventative measures and help in shifting individual and structural attention to existing gaps.

## Data Availability

The data analyzed in this study is subject to the following licenses/restrictions: The datasets presented in this article are not readily available due to ethical constraints. Requests to access these datasets should be directed to AH, a.hajek@uke.de.
