# Supplementary material for: Migration background, oral hygiene behavior and oral health literacy: findings based on a quota-based sample
Source: Front Oral Health. 2026 Apr 23;7:1791168. doi: 10.3389/froh.2026.1791168 (PMC13149372; doi:10.3389/froh.2026.1791168)
Supplement: Supplementary file 1 [file Table1.docx]

Supplementary Material

# Supplementary Tables

Supplementary Table 1. Oral hygiene behavior and oral health literacy – stratified by gender.

| Oral hygiene behavior | Men  (n=2,451, 49.0%) | Women  (n=2,540, 50.8%) | Diverse  (n=9, 0.2%) | P-value |
| --- | --- | --- | --- | --- |
|  |  |  |  |  |
| Frequency of use of electric toothbrush: N (%) |  |  |  | 0.04 |
| Not at all | 852 (34.8) | 918 (36.1) | 4 (44.4) |  |
| Once a week or less | 114 (4.7) | 115 (4.5) | 1 (11.1) |  |
| At least twice a week | 122 (5.0) | 106 (4.2) | 1 (11.1) |  |
| At least once a day | 594 (24.2) | 525 (20.7) | 1 (11.1) |  |
| At least twice a day | 769 (31.4) | 876 (34.5) | 2 (22.2) |  |
| Frequency of use of manual toothbrush: N (%) |  |  |  | <0.001 |
| Not at all | 728 (29.7) | 676 (26.6) | 3 (33.3) |  |
| Once a week or less | 289 (11.8) | 289 (11.4) | 2 (22.2) |  |
| At least twice a week | 195 (8.0) | 139 (5.5) | 0 (0.0) |  |
| At least once a day | 613 (25.0) | 587 (23.1) | 1 (11.1) |  |
| At least twice a day | 626 (25.5) | 849 (33.4) | 3 (33.3) |  |
| Frequency of use of fluoride gel: N (%) |  |  |  | <0.001 |
| Not at all | 1,443 (58.9) | 1,519 (59.8) | 6 (66.7) |  |
| Once a week or less | 392 (16.0) | 567 (22.3) | 1 (11.1) |  |
| At least twice a week | 207 (8.4) | 172 (6.8) | 1 (11.1) |  |
| At least once a day | 232 (9.5) | 150 (5.9) | 1 (11.1) |  |
| At least twice a day | 177 (7.2) | 132 (5.2) | 0 (0.0) |  |
| Frequency of use of interdental cleaning aids: N (%) |  |  |  | <0.001 |
| Not at all | 834 (34.0) | 541 (21.3) | 3 (33.3) |  |
| Once a week or less | 437 (17.8) | 489 (19.3) | 3 (33.3) |  |
| At least twice a week | 446 (18.2) | 538 (21.2) | 1 (11.1) |  |
| At least once a day | 494 (20.2) | 652 (25.7) | 2 (22.2) |  |
| At least twice a day | 240 (9.8) | 320 (12.6) | 0 (0.0) |  |
| Duration of tooth brushing: N (%) |  |  |  | 0.34 |
| About half a minute or less | 64 (2.6) | 48 (1.9) | 0 (0.0) |  |
| About one minute | 299 (12.2) | 294 (11.6) | 1 (11.1) |  |
| About two minutes | 1,102 (45.0) | 1,125 (44.3) | 4 (44.4) |  |
| About three minutes | 744 (30.4) | 843 (33.2) | 4 (44.4) |  |
| About four minutes or more | 242 (9.9) | 230 (9.1) | 0 (0.0) |  |
| Oral health literacy: N (%) |  |  |  | <0.001 |
| Excellent | 149 (6.1) | 128 (5.0) | 1 (11.1) |  |
| Very good | 492 (20.1) | 596 (23.5) | 1 (11.1) |  |
| Good | 1147 (46.8) | 1302 (51.3) | 4 (44.4) |  |
| Moderate | 606 (24.7) | 486 (19.1) | 3 (33.3) |  |
| Poor | 57 (2.3) | 28 (1.1) | 0 (0.0) |  |

Supplementary Table 2. Oral hygiene behavior and oral health literacy – stratified by age group.

| Oral hygiene behavior | 18 - 29 years (n=880, 17.6%) | 30 - 39 years (n=887, 17.7%) | 40 - 49 years (n=874, 17.5%) | 50 - 59 years (n=1,129, 22.6%) | 60 - 74 years (n=1,230, 24.6%) | P-value |
| --- | --- | --- | --- | --- | --- | --- |
|  |  |  |  |  |  |  |
| Frequency of use of electric toothbrush: N (%) |  |  |  |  |  | <0.001 |
| Not at all | 336 (38.2) | 234 (26.4) | 273 (31.2) | 401 (35.5) | 530 (43.1) |  |
| Once a week or less | 75 (8.5) | 53 (6.0) | 33 (3.8) | 33 (2.9) | 36 (2.9) |  |
| At least twice a week | 43 (4.9) | 67 (7.6) | 39 (4.5) | 36 (3.2) | 44 (3.6) |  |
| At least once a day | 171 (19.4) | 193 (21.8) | 207 (23.7) | 259 (22.9) | 290 (23.6) |  |
| At least twice a day | 255 (29.0) | 340 (38.3) | 322 (36.8) | 400 (35.4) | 330 (26.8) |  |
| Frequency of use of manual toothbrush: N (%) |  |  |  |  |  | <0.001 |
| Not at all | 198 (22.5) | 221 (24.9) | 231 (26.4) | 375 (33.2) | 382 (31.1) |  |
| Once a week or less | 101 (11.5) | 118 (13.3) | 124 (14.2) | 130 (11.5) | 107 (8.7) |  |
| At least twice a week | 66 (7.5) | 73 (8.2) | 80 (9.2) | 54 (4.8) | 61 (5.0) |  |
| At least once a day | 216 (24.5) | 226 (25.5) | 195 (22.3) | 245 (21.7) | 319 (25.9) |  |
| At least twice a day | 299 (34.0) | 249 (28.1) | 244 (27.9) | 325 (28.8) | 361 (29.3) |  |
| Frequency of use of fluoride gel: N (%) |  |  |  |  |  | <0.001 |
| Not at all | 489 (55.6) | 437 (49.3) | 488 (55.8) | 717 (63.5) | 837 (68.0) |  |
| Once a week or less | 204 (23.2) | 194 (21.9) | 193 (22.1) | 203 (18.0) | 166 (13.5) |  |
| At least twice a week | 67 (7.6) | 105 (11.8) | 77 (8.8) | 75 (6.6) | 56 (4.6) |  |
| At least once a day | 74 (8.4) | 83 (9.4) | 61 (7.0) | 66 (5.8) | 99 (8.0) |  |
| At least twice a day | 46 (5.2) | 68 (7.7) | 55 (6.3) | 68 (6.0) | 72 (5.9) |  |
| Frequency of use of interdental cleaning aids: N (%) |  |  |  |  |  | <0.001 |
| Not at all | 212 (24.1) | 151 (17.0) | 217 (24.8) | 337 (29.8) | 461 (37.5) |  |
| Once a week or less | 251 (28.5) | 178 (20.1) | 174 (19.9) | 169 (15.0) | 157 (12.8) |  |
| At least twice a week | 187 (21.2) | 212 (23.9) | 181 (20.7) | 228 (20.2) | 177 (14.4) |  |
| At least once a day | 162 (18.4) | 227 (25.6) | 200 (22.9) | 279 (24.7) | 280 (22.8) |  |
| At least twice a day | 68 (7.7) | 119 (13.4) | 102 (11.7) | 116 (10.3) | 155 (12.6) |  |
| Duration of tooth brushing: N (%) |  |  |  |  |  | <0.001 |
| About half a minute or less | 14 (1.6) | 16 (1.8) | 11 (1.3) | 32 (2.8) | 39 (3.2) |  |
| About one minute | 82 (9.3) | 77 (8.7) | 89 (10.2) | 132 (11.7) | 214 (17.4) |  |
| About two minutes | 382 (43.4) | 405 (45.7) | 393 (45.0) | 519 (46.0) | 532 (43.3) |  |
| About three minutes | 317 (36.0) | 292 (32.9) | 293 (33.5) | 353 (31.3) | 336 (27.3) |  |
| About four minutes or more | 85 (9.7) | 97 (10.9) | 88 (10.1) | 93 (8.2) | 109 (8.9) |  |
| Oral health literacy: N (%) |  |  |  |  |  | <0.001 |
| Excellent | 56 (6.4) | 100 (11.3) | 52 (5.9) | 49 (4.3) | 21 (1.7) |  |
| Very good | 200 (22.7) | 250 (28.2) | 222 (25.4) | 215 (19.0) | 202 (16.4) |  |
| Good | 386 (43.9) | 398 (44.9) | 433 (49.5) | 598 (53.0) | 638 (51.9) |  |
| Moderate | 228 (25.9) | 128 (14.4) | 155 (17.7) | 245 (21.7) | 339 (27.6) |  |
| Poor | 10 (1.1) | 11 (1.2) | 12 (1.4) | 22 (1.9) | 30 (2.4) |  |

Supplementary Table 3. Oral hygiene behavior and oral health literacy – stratified by educational level.

| Oral hygiene behavior | Primary education  (n=533, 10.7%) | Secondary education  (n=2,987, 59.7%) | Tertiary education  (n=1,480, 29.6%) | P-value |
| --- | --- | --- | --- | --- |
|  |  |  |  |  |
| Frequency of use of electric toothbrush: N (%) |  |  |  | <0.001 |
| Not at all | 262 (49.2) | 1,103 (36.9) | 409 (27.6) |  |
| Once a week or less | 23 (4.3) | 126 (4.2) | 81 (5.5) |  |
| At least twice a week | 24 (4.5) | 124 (4.2) | 81 (5.5) |  |
| At least once a day | 95 (17.8) | 705 (23.6) | 320 (21.6) |  |
| At least twice a day | 129 (24.2) | 929 (31.1) | 589 (39.8) |  |
| Frequency of use of manual toothbrush: N (%) |  |  |  | <0.001 |
| Not at all | 146 (27.4) | 894 (29.9) | 367 (24.8) |  |
| Once a week or less | 37 (6.9) | 323 (10.8) | 220 (14.9) |  |
| At least twice a week | 33 (6.2) | 168 (5.6) | 133 (9.0) |  |
| At least once a day | 146 (27.4) | 707 (23.7) | 348 (23.5) |  |
| At least twice a day | 171 (32.1) | 895 (30.0) | 412 (27.8) |  |
| Frequency of use of fluoride gel: N (%) |  |  |  | <0.001 |
| Not at all | 347 (65.1) | 1,866 (62.5) | 755 (51.0) |  |
| Once a week or less | 68 (12.8) | 570 (19.1) | 322 (21.8) |  |
| At least twice a week | 29 (5.4) | 206 (6.9) | 145 (9.8) |  |
| At least once a day | 45 (8.4) | 186 (6.2) | 152 (10.3) |  |
| At least twice a day | 44 (8.3) | 159 (5.3) | 106 (7.2) |  |
| Frequency of use of interdental cleaning aids: N (%) |  |  |  | <0.001 |
| Not at all | 232 (43.5) | 900 (30.1) | 246 (16.6) |  |
| Once a week or less | 62 (11.6) | 543 (18.2) | 324 (21.9) |  |
| At least twice a week | 75 (14.1) | 571 (19.1) | 339 (22.9) |  |
| At least once a day | 99 (18.6) | 679 (22.7) | 370 (25.0) |  |
| At least twice a day | 65 (12.2) | 294 (9.8) | 201 (13.6) |  |
| Duration of tooth brushing: N (%) |  |  |  | <0.001 |
| About half a minute or less | 26 (4.9) | 58 (1.9) | 28 (1.9) |  |
| About one minute | 90 (16.9) | 352 (11.8) | 152 (10.3) |  |
| About two minutes | 210 (39.4) | 1,371 (45.9) | 650 (43.9) |  |
| About three minutes | 142 (26.6) | 960 (32.1) | 489 (33.0) |  |
| About four minutes or more | 65 (12.2) | 246 (8.2) | 161 (10.9) |  |
| Oral health literacy: N (%) |  |  |  | <0.001 |
| Excellent | 24 (4.5) | 118 (4.0) | 136 (9.2) |  |
| Very good | 86 (16.1) | 603 (20.2) | 400 (27.0) |  |
| Good | 254 (47.7) | 1520 (50.9) | 679 (45.9) |  |
| Moderate | 147 (27.6) | 695 (23.3) | 253 (17.1) |  |
| Poor | 22 (4.1) | 51 (1.7) | 12 (0.8) |  |

Supplementary Table 4. Fit statistics (for adjusted logistic regression model with use of electric toothbrush as outcome)

Log-Lik Intercept Only: -3437.166 Log-Lik Full Model: -3378.281

D(4986): 6756.561 LR(10): 117.770

Prob > LR: 0.000

McFadden's R2: 0.017 McFadden's Adj R2: 0.013

Maximum Likelihood R2: 0.023 Cragg & Uhler's R2: 0.031

McKelvey and Zavoina's R2: 0.029 Efron's R2: 0.023

Variance of y*: 3.388 Variance of error: 3.290

Count R2: 0.573 Adj Count R2: 0.044

AIC: 1.357 AIC*n: 6784.561

BIC: -35710.164 BIC': -32.599
